# A Systematic Review and Meta-Analysis: Evaluation of the β-Human Papillomavirus in Immunosuppressed Individuals with Cutaneous Squamous Cell Carcinoma

**DOI:** 10.37796/2211-8039.1110

**Published:** 2020-12-01

**Authors:** Mazaher Ramezani, Farideh Baharzadeh, Afshin Almasi, Masoud Sadeghi

**Affiliations:** a Molecular Pathology Research Center, Imam Reza Hospital, Kermanshah University of Medical Sciences, Kermanshah, Iran; b Students Research Committee, Kermanshah University of Medical Sciences, Kermanshah, Iran; c Department of Biostatistics, School of Health, Kermanshah University of Medical Sciences, Kermanshah, Iran; d Medical Biology Research Center, Kermanshah University of Medical Sciences, Kermanshah, Iran

**Keywords:** skin cancer, β-HPV, SCC, prevalence, immunosuppression, meta-analysis

## Abstract

**Background:**

Some types of beta-human papillomavirus (β-HPV) may be one of the probable causes of squamous cell carcinoma (SCC) in transplant recipients. β-HPVs are linked to SCC in the literature with small number of subjects.

**Aim:**

Herein, the first meta-analysis was carried out on the association between β-HPVs and cutaneous SCC in immunosuppressed patients.

**Methods:**

A systematic search was carried out in the PubMed and Scopus databases up to December 2018. The odds ratio (OR) were calculated by RevMan 5.3 software and the event rate (ER) by Comprehensive Meta-Analysis 2.0 software with a 95% confidence interval (CI).

**Results:**

A total of 1250 records were identified through the two databases, but at last eleven studies were included in the meta-analysis that they were published from 1989 to 2018. The results showed a significantly high prevalence of β-HPVs in cutaneous SCC patients (ER = 69.1%; 95%CI: 58.7%, 77.8%). In addition, the prevalence of overall β-HPVs and β-HPVs of 5, 8, 9, 17, 49, 75, and 76 in immunosuppressed cutaneous SCC patients was significantly higher compared with controls.

**Conclusions:**

The findings of the present meta-analysis support the hypothesis that β-HPV may play a role in cutaneous SCC development in immunosuppressed individuals.

## 1. Introduction

Among skin cancers, cutaneous SCC (cSCC) is a quite common malignant proliferation of the epithelial layers with an aggressive behavior and possible metastasis [1]. The risk factors for the SCC development are represented by fair skin, intense sun-exposure, history of sunburns, immunosuppression and beta-human papillomavirus (β-HPV) infection. Indeed, one of the probable causes of SCC is the HPV [2, 3]. HPVs are a great and various group of over 170 subsets with 5 main known HPV genera, including Alpha, Beta (β), Gamma, Mu and Nu papillomavirus [4, 5]. While other HPV genera contribute to the formation of verruca vulgaris, condyloma acuminate, and various types of anogenital cancers [6, 7], the β-genus appears to be involved in human cutaneous carcinogenesis and in promoting non-melanoma skin cancer development in immunosuppressed patients [8]. Among β-HPVs, HPVs 5 and 8 seem to have a potential role in warts that may culminate in SCC formation [9]. Due to the high incidence of SCC in organ transplant recipients in comparison with the general population as well as the similarity of their symptoms, clinical behavior and epidemiology with other virus-induced cancers (i.e. Kaposi's sarcoma), some studies have focused on their origin [10]. Among them, several studies have demonstrated that a further increase in the SCC appearance in transplant recipients is correlated with a significant degree of β-HPV detection [11, 12]. It was reported that 90% of the SCC lesions in Epidermodysplasia Verruciformis patients - a rare genodermatosis determined by multiple skin cancers on sun-exposed sites-were found to increase the likelihood of viral carcinogenicity [13]. Recently, the association between β-HPV and SCC in immunosuppressed patients has been studied in several epidemiological studies with controversial results [14, 15]. Considering the hypothesis that β-HPV may play a role in cSCC development in immunosuppressed individuals, this meta-analysis was carried out to explore an association between β-HPVs and cSCC in immunosuppressed patients.

## 2. Materials and Methods

### 2.1. Identification of studies

A systematic search was carried out in the PubMed and Scopus databases up to December 2018 without any restrictions. To retrieve the studies, one of authors (M.S) searched among two databases with the search strategy of (“human papillomavirus” or “HPV” or 00β_-HPV”_ or “beta-HPV”) and (“cutaneous squamous cell carcinoma” or “cutaneous SCC” or “skin squamous cell carcinoma” or “skin SCC” or “cSCC” or “nonmelanoma skin neoplasms” or “nonmelanoma skin cancer” or “squamous cell cancer” or “squamous cell neoplasm”). In addition, the citations of the retrieved studies in relation to the topic of our meta-analysis were reviewed to ensure that no studies were missed. After that, other authors (M.R and F.B) assessed the relevant articles based on the titles and abstracts. Subsequently, the articles with the full-text meeting the criteria were screened. After screening, the reasons for exclusion were written for the studies removed and another author (A.A) resolved the disagreements between the authors.

### 2.2. Eligibility criteria

The authors used the mentioned criteria for selecting the studies. We included: 1) all types of studies; 2) inclusion of immunosuppressed patients of any age and gender; 3) diagnosis of cSCC established according to clinical and/or histologic criteria, and 4) studies reporting the prevalence of β-HPVs in cSCC patients. On the contrary, we excluded studies with irrelevant or unavailable data, studies including noncutaneous SCC, studies including unspecified nonmelanoma skin cancer, animal studies, duplicate studies, and conference papers, case reports, and reviews.

### 2.3. Data abstraction

The data of the studies entered to the analysis including first name of first author, publication year, research area, study design, number of individuals and β-HPV detection methods/subtypes, were independently extracted and analyzed by three authors (M.S, M.R and F.B).

### 2.4. Statistical analysis

The values of odds ratio (OR) were computed by Review Manager version 5.3 software and the event rate (ER) by Comprehensive Meta-Analysis-version 2.0 software with a 95% confidence interval (CI). To estimate the pooled OR significance, the Z test was applied with a *p*-value (2-sided) < 0.05. In addition, the I^2^ statistic was applied to estimate heterogeneity that if P > 0.1 (I^2^<50%), there was a significant heterogeneity and in this state, we used the fixed-effects model; otherwise, the random-effects model was used. The Funnel plots were analyzed with both Egger's and Begg's tests that P < 0.05 (two-sided) showed the significant publication bias. To estimate the stability of the pooled data, the sensitivity analyses (“cumulative analysis” and “the removal of one study”) were applied.

## 3. Results

### 3.1. Study selection

**Fig. 1:**
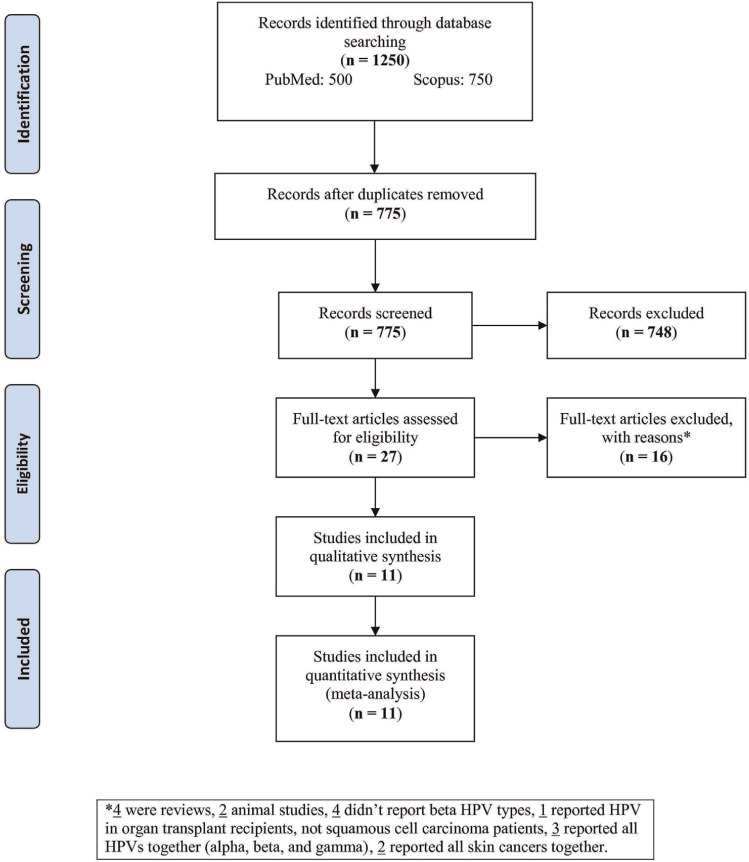
Flow-chart of the study selection.

A total of 1250 records were identified through the two databases that after removing the duplicates, 775 records were screened, among which 748 irrelevant records were excluded (Fig. 1). Then, 27 full-texts were evaluated, from among which 16 full-texts were excluded with reasons (four reviews, two animal studies, four studies not reporting β-HPV types, one reporting HPV in organ transplant recipients not affected by squamous cell carcinoma, three reporting all HPV genera altogether (alpha, beta, and gamma), and two reporting different skin cancers altogether). At last, eleven studies were entered to the analysis.

### 3.2. Features of studies

Table 1 is illustrated the features of eleven studies included in the meta-analysis published from 1989 to 2018. Four studies were presented from United Kingdom [16-19], two from the Netherlands [20,21], one from Scotland [22], one from the United States of America [23], one from Ireland [24], one from Germany [25] and one was a multicenter study (Queensland, Australia, and Italy) [26]. Six studies were uncontrolled [16,19,22-25], two were case-control [20,26] and three were cohort [17,18,21] studies. The detection methods of β-HPVs were the Polymerase Chain Reaction (PCR) for eight studies [16,18,19,22-26], serology for two studies [17,21] and PCR and serology together for two studies [20].

### 3.3. Meta-analysis

**Fig. 2:**
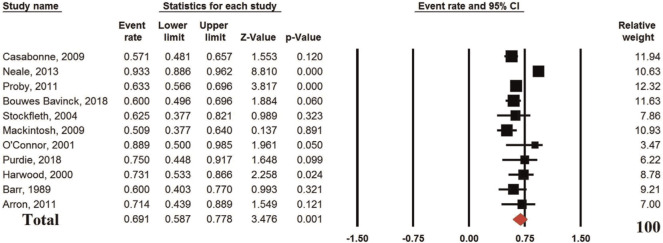
Event rate of the prevalence of b-HPVs in immunosuppressed patients with cSCC.

The pooled analysis of eleven studies reporting the prevalence of β-HPVs in immunosuppressed cSCC patients showed an ER of 69.1% (95%CI: 58.7%, 77.8%; *p* = 0.001; I^2^ 87.7% (P_h_ or P_heterogeneity_<0.0001) (Fig. 2). The results showed a significantly high prevalence of β-HPVs in cSCC in immunosuppressed patients.

**Fig. 3:**
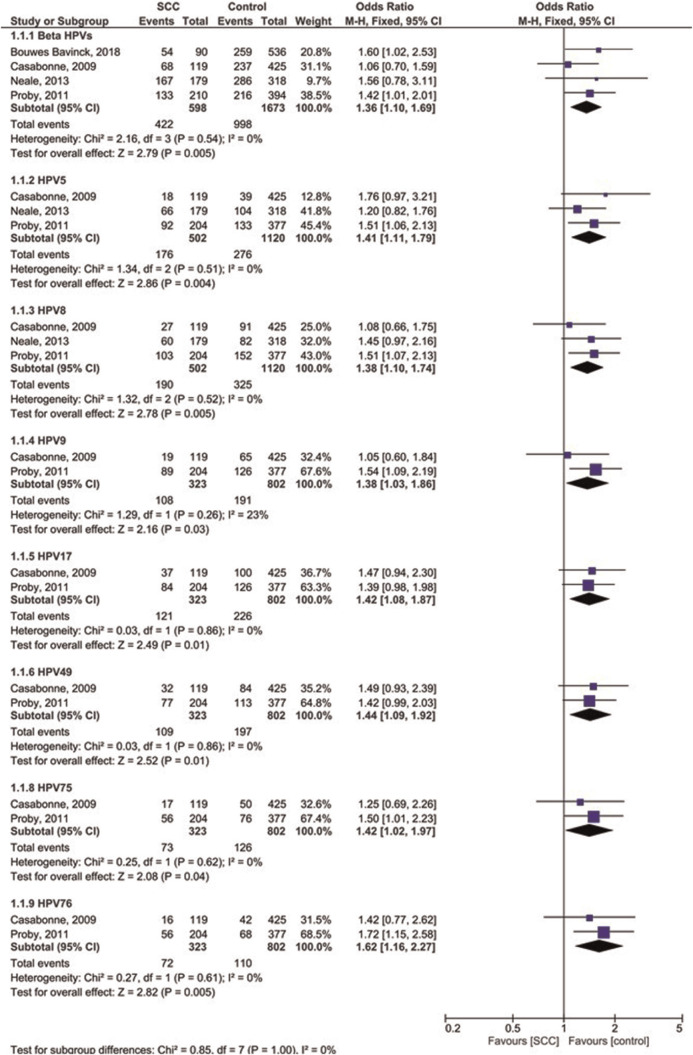
Forest plot of odds ratio of prevalence of some b-HPVs in immunosuppressed patients with SCC compared to healthy controls.

**Fig. 4:**
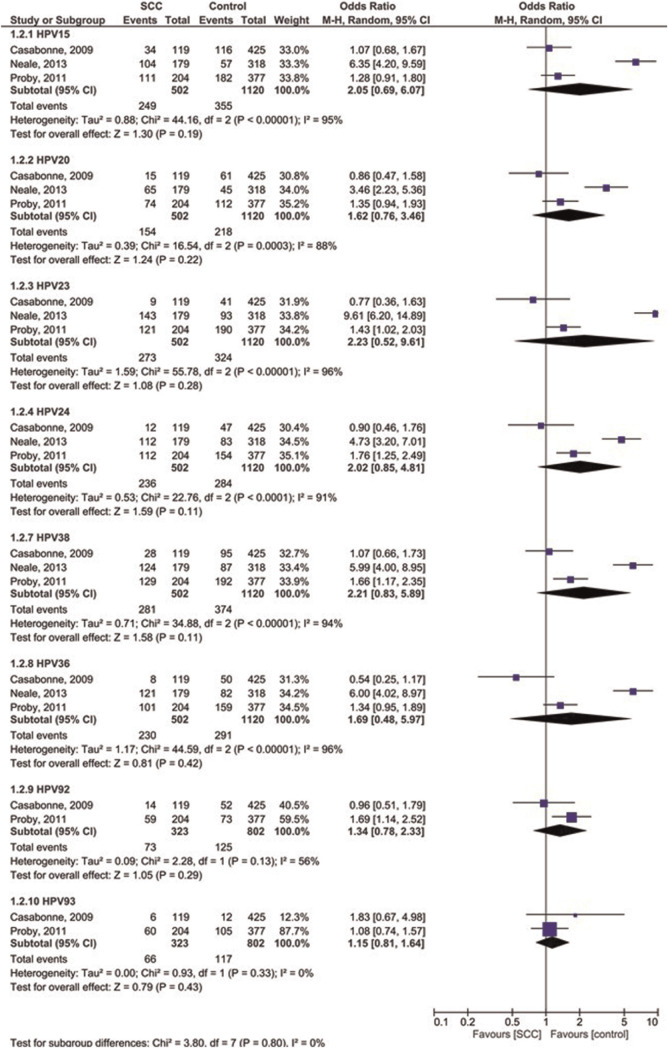
Forest plot of odds ratio of prevalence of other b-HPVs in immunosuppressed patients affected by SCC compared to healthy controls.

Four case-control studies were analyzed for OR of β-HPVs in immunosuppressed cutaneous patients affected by SCC compared to controls (Figs. 3 and 4). The OR was 1.36 for overall b-HPVs [95%CI: 1.10, 1.69; *p* = 0.005] without heterogeneity, 1.41 for HPV5 [95%CI: 1.11, 1.79; *p* = 0.004] without heterogeneity, 1.38 for HPV8 [95%CI: 1.10, 1.74; *p* = 0.005] without heterogeneity, 1.38 for HPV9 [95%CI: 1.03, 1.86; *p* = 0.03; I^2^ = 23% (P_h_ = 0.26)], 1.42 for HPV17 [95% CI: 1.08, 1.87; *p* = 0.01] without heterogeneity, 1.44 for HPV49 [95%CI: 1.09, 1.92; *p* = 0.01] without heterogeneity, 1.42 for HPV75 [95%CI: 1.02, 1.97; *p* = 0.04] without heterogeneity, and 1.62 for HPV76 [95%CI: 1.16, 2.27; *p* = 0.005] without heterogeneity. In addition, the OR was 2.05 for HPV15 [95%CI: 0.69, 6.07; *p* = 0.19; I^2^ = 95% (P_h_ < 0.00001)], 2.23 for HPV23 [95%CI: 0.52, 9.61; *p* = 0.28; I^2^ = 96% (P_h_ < 0.00001)], 2.21 for HPV38 [95%CI: 0.83, 5.89; *p* = 0.11; I^2^ = 94% (P_h_ < 0.00001)], 1.34 for HPV92 [95%CI: 0.78, 2.33; *p* = 0.29; I^2^ = 56% (P_h_ = 0.13)], 1.62 for HPV20 [95%CI: 0.76, 3.46; *p* = 0.22; I^2^ = 88% (P_h_ = 0.0003)], 2.02 for HPV24 [95%CI: 0.85, 4.81; *p* = 0.11; I^2^ = 91% (P_h_ < 0.0001)], 1.69 for HPV36 [95% CI: 0.48, 5.97; *p* = 0.42; I^2^ = 96% (P_h_ < 0.00001)] and 1.14 for HPV93 [95%CI: 1.80, 1.63; *p* = 0.45] without heterogeneity. The results showed that the prevalence of overall β-HPVs and β-HPVs of 5, 8, 9, 17, 49, 75, and 76 was significantly higher in immunosuppressed SCC patients than in controls.

### 3.4. Sensitivity analysis

Two sensitivity analyses mentioned in methods were performed on the prevalence of β-HPVs in immunosuppressed patients with SCC. We found that the pooled ER did not alter; therefore, these results confirmed the stability of initial pooled data.

### 3.5. Publication bias

**Fig. 5:**
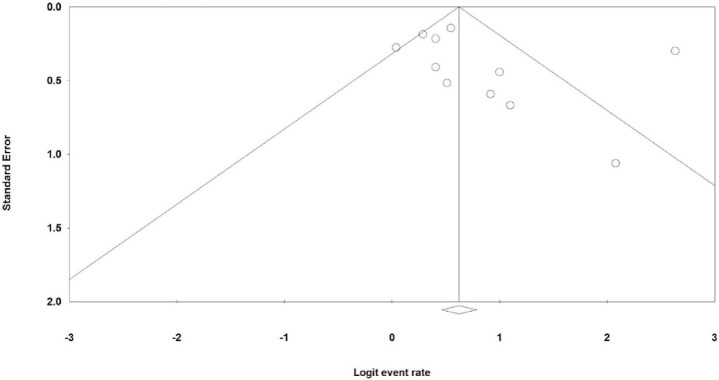
Funnel plot of the prevalence of b-HPVs in immunosuppressed patients with SCC.

With regard to publication bias, Fig. 5 illustrates the funnel plot of the prevalence of β-HPVs in immunosuppressed patients affected by SCC. The Begg's test revealed a bias between the studies (*p* = 0.035), but no Egger's test (*p* = 0.340).

## 4. Discussion

SCC is usually a very rapidly growing dangerous tumor with the proliferation of keratinocytes [27,28]. Renal transplant recipients have an elevated incidence of HPV-related cancers [29-33]. The beta genus comprises more than 50 β-HPVs [34]. Some studies have linked β-HPVs to SCC, but many studies have considered a small number of subjects and/or samples and others have been uncontrolled. The present meta-analysis evaluated the ER of β-HPVs in immunosuppressed patients with SCC and also the OR of β-HPVs among these patients in comparison to controls. The results showed that the prevalence of β-HPVs in such patients was significantly higher and also the OR of some β-HPV genotypes was significantly higher in patients than in controls (overall β-HPVs and β-HPVs of 5, 8, 9, 17, 49, 75, and 76).

The studies have shown a high detection of HPV in both precancerous lesions and SCC in renal transplant recipients (81% to 91%) [35,36]. The β-HPV prevalence in the studies included in the present meta-analysis was ranged from 50.9% to 93.3%; similarly, among uncontrolled studies, it varied from 50.9% to 88.9%. Among case-control [20,26] and cohort studies [17,21], including immunosuppressed individuals with SCC, one cohort study [21] based on serology and one case-control study [20] including the individuals with β-HPV antibodies showed a significantly increased risk. Two other studies [17,26] failed to illustrate significantly a difference between the patients and controls. The controversy between the results and the wide range of outcomes may be due to using different methods with different sensitivity and specificity.

A previous meta-analysis [37] showed that cSCCs were more probably to carry β-HPV genotypes compared to healthy skin, and there was an increase in β-HPV prevalence in tumors of immunosuppressed subjects in comparison to immunocompetent subjects. Concerning differences in β-HPV prevalence among different groups of patients, Harwood et al. [16] reported a different detection rate of 84% versus 27% between two groups of patients (immunosuppressed versus immunocompetent subjects), which was confirmed by Stockfleth et al. subsequently (75% vs. 37%) [18]. However, another study [20] did not detect differences (51% versus 52%) in viral detection among paraffin- embedded tumors from immunosuppressed and immunocompetent subjects. The younger age of immunosuppressed patients than immunocompetent individuals was suggested to explain such a result. Accordingly, several studies [23,38-40] reported that immunosuppression and older age had an association with the viral load and higher prevalence of β-HPV. In fact, it can be concluded that considering the age of the patient along with immune status is important in a correct interpretation of the results and predicting the outcome. Moreover, some studies have confirmed HPVs 5 and 8 to be more frequently detected in the SCC from organ transplant recipients [25,41,42]. Two studies [18,43] showed that β-HPV was detected in a greater proportion of frozen samples compared to paraffin-embedded specimens. A study [44] using Southern hybridization and type-specific PCR failed to detect any HPV 5 and 8 in 30 cSCC samples. Another study [45] did not identify any HPV-DNA in 28 non-genital SCCs from immunosuppressed renal allograft recipients. The methodical differences and the different clinical specimens can affect improved β-HPV detection [25]. Therefore, the used techniques (with their own different sensitivities) can explain the different results, and it is also mandatory to pay attention to such issues in β-HPV detection while analyzing the data. Another issue to be considered is the productive status of the virus *versus* the higher DNA (or even its small fragments) detection rate since this is of primary importance when investigating the role of β-HPVs in cutaneous carcinogenesis.

Moreover, the studies reported that the sun-exposed samples showed higher β-HPV DNA prevalence in both immunosuppressed and immunocompetent individuals, which can help the theory of a powerful interaction between ultraviolet (UV) radiation and β-HPV presence [46-48]. Therefore, both immunosuppression and UV radiation may elevate the β-HPV activity that is able to promote the development of cancer [25,49].

The present meta-analysis suffers from several significant limitations. First, few studies reported. Second, most studies included a few number of patients. Third, different methods were used for the detection of β-HPVs. Fourth; there were differences in the detected β-HPV genotypes. These limitations and also the role of age, UV radiation, and geographical origin of cases could affect the obtained results in terms of β-HPV prevalence and also create a high heterogeneity across the included studies.

## 5. Conclusions

The findings of the present meta-analysis support the hypothesis that β-HPV may play a main role in the cSCC development in immunosuppressed subjects. The prevalence of β-HPVs in these patients was 69.1%, and several genotypes (overall β-HPVs and β-HPVs of 5, 8, 9, 17, 49, 75, 76, and 93) were associated with an elevated risk of developing cSCC in immunosuppressed subjects compared to healthy controls. Notwithstanding, it should be noted that demographic and environmental factors can affect the β-HPV prevalence. We believe that further studies are currently needed to include a great number of participants from different geographic areas. In order to confirm our results, it is also important to notice the β-HPV detection methods and considered genotypes.

## Figures and Tables

**Table 1:** Characteristics of the studies included in the meta-analysis.

First author, year publication	Country	Design/Population	β-HPV Detection Methods/Subtypes
Barr, 1989 [22]	Scotland	Uncontrolled	PCR
		Case: 25	HPV type 5/8
Harwood, 2000 [16]	United Kingdom	Uncontrolled	PCR
		Case: 26	All beta HPV types
Arron, 2011 [23]	United States of America	Uncontrolled	PCR
		Case: 14	All beta HPV types
O'Connor, 2001 [24]	Ireland	Uncontrolled	PCR
		Case: 9	All beta HPV types
Stockfleth, 2004 [25]	Germany	Uncontrolled	PCR
		Case: 16	HPV types 5, 8
Casabonne, 2009 [17]	United Kingdom	Cohort	Serology
		Case: 119	HPV types 5, 8, 9, 15, 17, 20, 23, 24, 36, 38,
		Control: 425	49, 75, 76, 92, 93, 96
Mackintosh, 2009 [18]	United Kingdom	Cohort	PCR
		Case: 53	HPV types 5, 8, 9, 12, 14, 15, 17, 19e25, 36 −38, 47, 49, 75, 76, 80, 92, 93 and 96
Proby, 2011 [20]	The Netherlands	Case-control	PCR & Serology
		Case: 204	HPV types 5, 8, 9, 15, 17, 20, 23, 24, 36, 38,
		Control: 377	49, 75, 76, 92, 93, 96
Neale, 2013 [26]	Queensland, Australia, and Italy	Case-control	PCR
		Case: 179	HPV types 5, 8, 15, 20, 23, 24, 36, 38
		Control: 318	
Bouwes Bavinck, 2018 [21]	The Netherlands	Cohort	Serology
		Case: 90	All beta HPV types
		Control: 536	
Purdie, 2018 [19]	United Kingdom	Uncontrolled	PCR
		Case: 12	All beta HPV types

Abbreviations: PCR, Polymerase chain reaction; HPV, Human papillomavirus.
